# Late-Onset Non-HLH Presentations of Growth Arrest, Inflammatory Arachnoiditis, and Severe Infectious Mononucleosis, in Siblings with Hypomorphic Defects in *UNC13D*

**DOI:** 10.3389/fimmu.2017.00944

**Published:** 2017-08-09

**Authors:** Paul Edgar Gray, Bella Shadur, Susan Russell, Richard Mitchell, Michael Buckley, Kerri Gallagher, Ian Andrews, Kevin Thia, Joseph A. Trapani, Edwin Philip Kirk, Ilia Voskoboinik

**Affiliations:** ^1^Department of Immunology and Infectious Diseases, Sydney Children’s Hospital, Randwick, NSW, Australia; ^2^Kids Cancer Centre, Sydney Children’s Hospital, Randwick, NSW, Australia; ^3^Genetics Laboratory, South Eastern Area Laboratory Services, Randwick, NSW, Australia; ^4^Department of Immunology, Royal Prince Alfred Hospital, Sydney, NSW, Australia; ^5^Department of Neurology, Sydney Children’s Hospital, Randwick, NSW, Australia; ^6^Cancer Cell Death Laboratory, Cancer Immunology Research, Peter MacCallum Cancer Centre, Melbourne VIC, Australia; ^7^Centre for Clinical Genetics, Sydney Children’s Hospital, Randwick, NSW, Australia; ^8^Killer Cell Biology Laboratory, Cancer Immunology Research, Peter MacCallum Cancer Centre, Melbourne, VIC, Australia

**Keywords:** cytotoxic lymphocytes, HLH, immunodeficiency, hematology, pathology

## Abstract

Bi-allelic null mutations affecting *UNC13D, STXBP2*, or *STX11* result in defects of lymphocyte cytotoxic degranulation and commonly cause familial hemophagocytic lymphohistiocytosis (FHL) in early life. Patients with partial loss of function are increasingly being diagnosed after presenting with alternative features of this disease, or with HLH later in life. Here, we studied two sisters with lymphocyte degranulation defects secondary to compound heterozygote missense variants in *UNC13D*. The older sibling presented aged 11 with linear growth arrest and delayed puberty, 2 years prior to developing transient ischemic attacks secondary to neuroinflammation and hypogammaglobulinemia, but no FHL symptoms. Her geno-identical younger sister was initially asymptomatic but then presented at the same age with severe EBV-driven infectious mononucleosis, which was treated aggressively and did not progress to HLH. The sisters had similar natural killer cell degranulation; however, while cytotoxic activity was moderately reduced in the asymptomatic patient, it was completely absent in both siblings during active disease. Following allogeneic bone marrow transplantation at the age of 15, the older child has completely recovered NK cell cytotoxicity, is asymptomatic, and has experienced an exceptional compensatory growth spurt. Her younger sister was also successfully transplanted and is currently disease free. The current study reveals previously unappreciated manifestations of FHL in patients who inherited hypomorphic gene variants and also raises the important question of whether a threshold of minimum NK function can be defined that should protect a patient from serious disease manifestations such as HLH.

## Introduction

The autosomal recessive disorder familial hemophagocytic lymphohistiocytosis (FHL) results from mutations in the various genes that regulate cytotoxic lymphocyte degranulation and their ability to kill virus-infected cells *PRF1, UNC13D, STX11*, and *STXBP2* genes (1, 2). Recently, it has become apparent that some patients with these defects can present with atypical clinical manifestations and/or delayed disease onset (3–5). One hypothesis is that these patients possess genotypes that encode proteins with some residual cytotoxic activity of NK cells; however, because most patients present with serious clinical illness that can secondarily reduce NK cell function, this association has been difficult to demonstrate. Here, we studied two sisters with FHL due to missense mutations in *UNC13D*, formally demonstrating the relationship between a hypomorphic immune phenotype and attenuated clinical manifestations. We also show that the residual NK cell cytotoxicity associated with hypomorphic mutations is further reduced during episodes of severe systemic illness.

## Case Report

**Figure 1:**
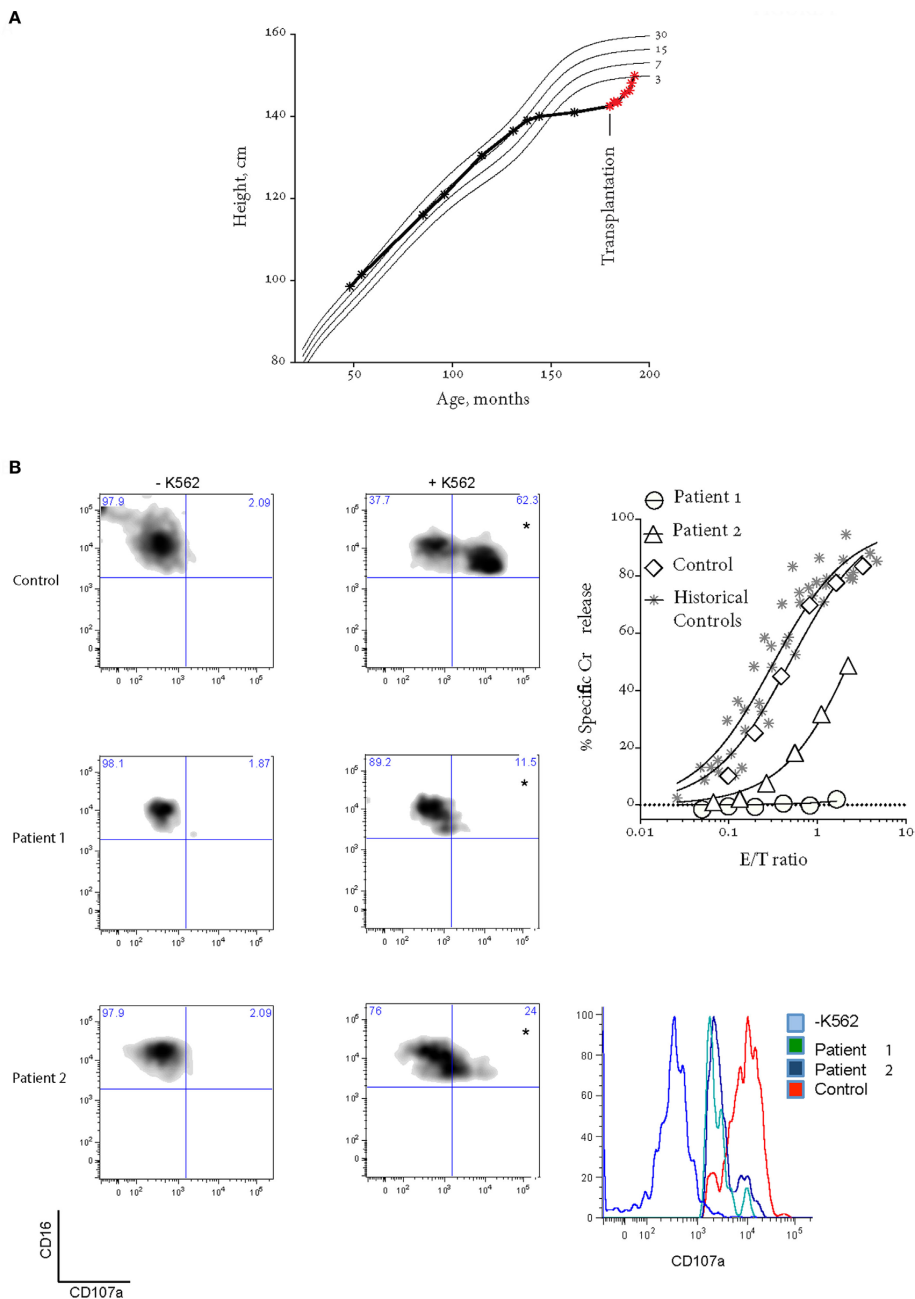
Patient 1 prior to bone marrow transplantation (BMT). **(A)** Patient 1 suffered growth arrest between the ages of 11 and 15. Following BMT, the patient has grown by over 8 cm. **(B)** Bi-allelic mutations in *UNC13D* result in impaired CD107a degranulation (on the left) and NK cell cytotoxicity using a standard 4-h ^51^Cr release assay (on the right). Histogram on the bottom right shows CD107a^+^ NK cells from quadrants labeled with “*”. In the ^51^Cr release assay, *E*/*T* ratio was calculated based on %NK cells (CD3^−^CD16^+^CD56^+^) in peripheral blood mononucleated cell (PBMC). “Historical controls” include seven unrelated healthy donors assessed at different times over a period of 2 years. “Control” is a healthy donor who provided the blood at the same time as the patients. Freshly isolated PBMCs were cultured overnight in 100 U/mL IL2.

Written informed consent was obtained from the participant for the publication of this case report. Patient 1 is a now 16-year-old Caucasian girl (from non-consanguineous parents) suffered linear growth arrest from the age of 11 and was delayed going into puberty until after treatment aged 15 (Figure 1A). She presented to our service aged 13 with headaches and 6 episodes of transient hemiparesis affecting the right side of her body and face. This was thought secondary to raised intracranial pressure (ICP > 50 mmHg), which in turn was secondary to CNS inflammation [CSF protein = 1.03 g/L (reference range 0.15–0.45 g/L), neopterins = 544.51 nmol/L (6–30 nmol/L), and positive oligoclonal bands]. MRI, PET scan, and angiographic imaging of brain were normal, and a screen of neurotropic viruses was negative. She was diagnosed with inflammatory arachnoiditis and was noted to have an associated sensory neuropathy.

Further investigation showed dysgammaglobulinemia with undetectable IgA and elevated IgG and IgM, associated with low B-cell numbers = 0.02 × 10^9^/L (0.2–0.6 × 10^9^/L)(6). The presence of a pervasive inflammatory disease with dysgammaglobulinemia prompted consideration of atypical FHL (6, 7), and we assessed NK cell function and then conducted mutation screening as follows. NK cell degranulation and target cell lysis were assessed following incubation of isolated peripheral blood mononucleated cells (PBMCs) with K562 cells: the former through CD107a expression on CD3^−^CD16^+^CD56^+^ NK cells, the latter using a 4-h ^51^Cr release assay (standardized for %NK). Healthy donor PBMC were isolated at the same time and used as controls. Freshly isolated PBMC were cultured for 20–24 h in the absence or presence of 100 U/mL IL-2. Mutation screening of patient *PRF1, UNC13D, STX11*, and *STXBP2* exon sequences and intron-exon boundaries was performed by Centogene AG (Rostock, Germany). Variant frequency was obtained from the EXAC database http://biorxiv.org/content/early/2015/10/30/030338, while *in silico* analysis employed a variety of tools including SIFT, MutationTaster, Provean, PhyloP, and CADD.

The patient’s NK cell cytotoxicity was undetectable and NK degranulation was severely reduced (Figure 1B). Genetic screening revealed rare compound heterozygous variants in *UNC13D*: c.[1240C>T] leading to p.Arg414Cys and c.[2753C>A] leading to p.Ala918Cys both of which are predicted to be pathogenic by *in silico* analysis (Table 1). The c.1240C>T was previously reported in an 11-year-old Turkish boy (born to consanguineous parents) with EBV-associated neurologic FHL (8), which mirrored somewhat the patient presented here, and Arg414 is invariably conserved. While the [2753C>A] variant has not previously been reported, the residue Ala918 is invariably conserved as far as in *C. elegans* (Ce_F54G2.1; gi: 17568145; 25% homology with human MUNC13-4), and the mutation was predicted to be detrimental (Table 1).

**Table 1:** *In silico* prediction analysis of *UNC13D* mutations.

	c.[1240C>T] p.Arg414Cys	c.[2753C>A] p.Ala918Asp
Previous clinical reports	HLH in homozygous state	None
gnomAD allele frequency	1 in 245878	Not previously reported
Evolutionary conservation	Highly conserved, including *C. elegans*	Highly conserved, including *C. elegans*
SIFT	Damaging	Damaging
MutationTaster	Disease causing	Disease causing
Provean	Damaging (−7.47)	Damaging (−3.93)
CADD	32 (0.06%)	14.5

**Figure 2:**
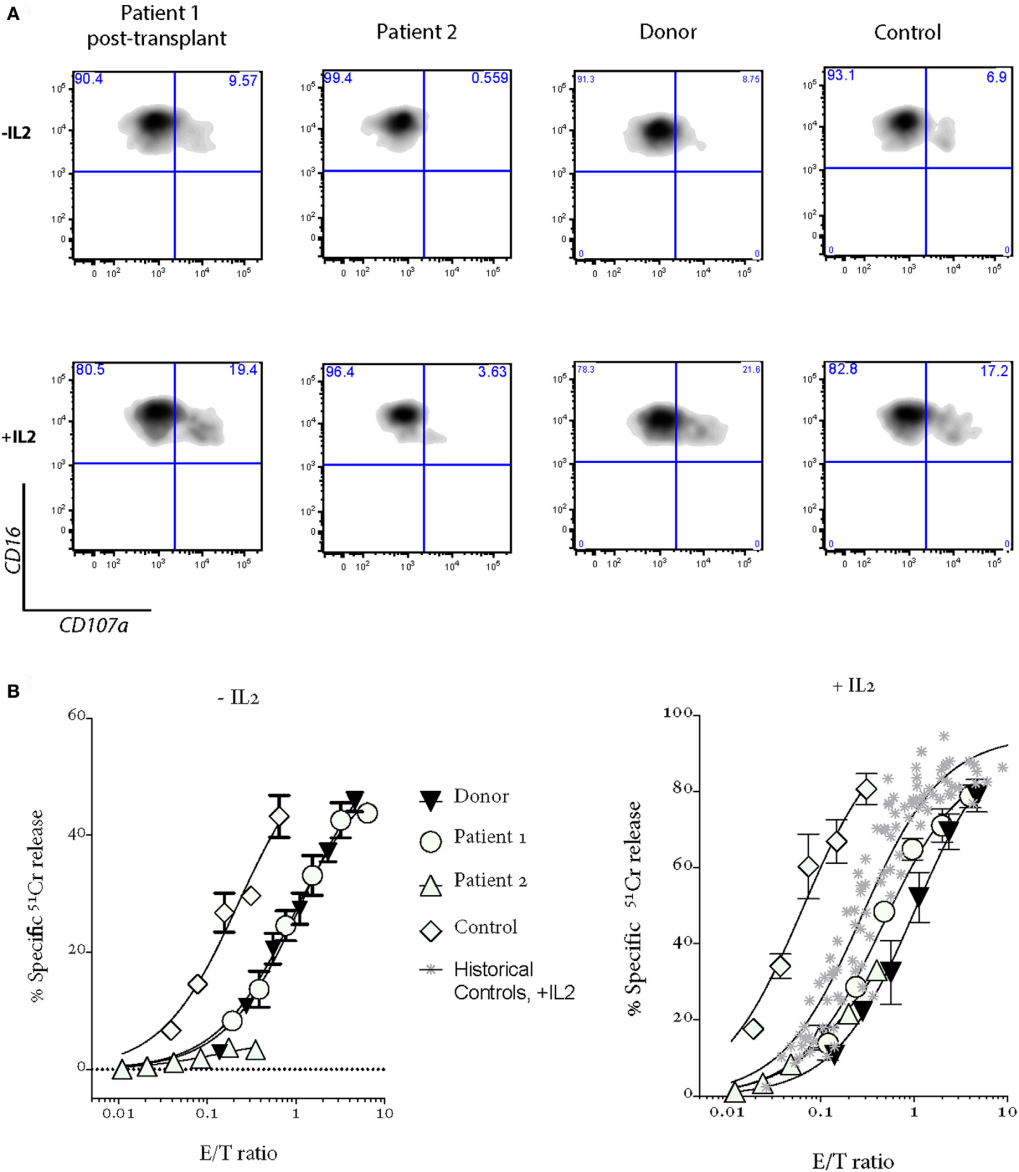
Patient 1 NK cell cytotoxicity and degranulation show a complete recovery after bone marrow transplantation; NK cell cytotoxicity and degranulation are severely impaired in Patient 2 after a severe EBV-driven mononucleosis. Shown are **(A)** degranulation assays and **(B)** 4-h ^51^Cr release assays. “Donor” is a sibling of Patients 1 and 2, who carries a mono-allelic *UNC13D* mutation; “Control” is a healthy donor who provided the blood at the same time as Patients 1 and 2; “Historical controls” are from 16 healthy donors, including those shown in Figure 1B. PBMCs were incubated overnight without (−IL2) or with 100 U/mL IL2 (+IL2).

Patient 1 progressed to allogeneic bone marrow transplantation (BMT) from a 10/10 matched unrelated donor, using a reduced intensity conditioning (RIC) regimen including intermediate timing of Alemtuzemab (9), and at 14 months posttransplant is asymptomatic and has grown over 10 cm (Figure 1A). She has 100% donor chimerism and complete recovery of NK cell activity (Figure 2A). Unfortunately, there was never an opportunity to measure Patient 1’s NK cell cytotoxicity when she was free of disease.

Her younger sister (Patient 2), who was asymptomatic at the time of the older child’s diagnosis, was found on screening to have inherited the same variants in *UNC13D*. Her NK cell cytotoxicity was approximately 20% of control (Figure 1B), suggesting that Patient 1 might also have had measurable (but reduced) NK function when well. In support of this notion, while there was a substantial difference in the number of degranulating NK cells between the siblings, the extent of degranulation of individual cells was almost indistinguishable, consistent with their identical *UNC13D* genotype (Figure 1B, *histogram*). Patient 2 was planned for preventative stem cell transplant from a third unaffected HLA-matched sibling donor (MSD), who carries only the c.[2753 C>A] variant of *UNC13D*. Because of concerns that cells from a heterozygote donor might complicate partial donor chimerism posttransplant (e.g., if the mutation was dominant negative), we tested her NK cell function and found only minor reduction in activity that was consistent with her genotype (Figure 2, see “Donor”) and with an earlier report on carriers of pathogenic FHL-causing mutations (10). However, prior to the transplant taking place, Patient 2 presented aged 9 with severe EBV-driven infectious mononucleosis, complicated by massive tonsillar hypertrophy requiring intubation for airway protection. Similar to Patient 1, the cytotoxic activity of her NK cells was severely reduced, but it recovered in the presence of IL2 (Figure 2); at the same time, NK cell degranulation remained marginal under both conditions. Previously, it was shown that IL2 partly restored the function of NK cells in patients with hypomorphic perforin mutations (11). Patient 2 was treated with a combination of high dose corticosteroids, anti-CD20 therapy with rituximab and cyclosporine, and did not develop HLH. At the time of writing, she is 45 days post-RIC BMT from her MSD and is 100% donor engrafted.

## Discussion

By examining genotype–phenotype relationships of FHL-causing mutations, it is now clear that some patients do not meet the criteria for FHL at disease onset and often present with atypical immune dysregulation, or HLH much later than was previously considered likely (4, 12, 13). Localized or organ-specific inflammation, particularly affecting the CNS, is increasingly being reported as an initial presentation of FHL (3, 14–16), as are vasculitis (15, 17, 18), granulomatous pneumonitis (19, 20), and arthritis (17, 21). The index case discussed here had previously unreported presentations of FHL-causing gene deficiency, namely: severe growth arrest and delayed puberty, beginning at the age of 11, followed by raised ICP secondary to presumed inflammatory arachnoiditis 2 years later. All the symptoms were completely reversed following transplantation, with compensatory growth spurt being particularly remarkable. The fact that the sisters presented at the same age is also noteworthy, with some other reports suggesting consistency in the age of presentation (1), while others report members of the same family with bi-allelic FHL gene mutations presenting with variable phenotypes and distinct primary symptoms (11, 12). The latter suggests a role for additional genetic modifiers or, potentially, environmental factors.

It is important to point out the ongoing difficulty in the era of genetic screening, which commonly reveals multiple irrelevant variants, of confirming the biological importance of novel missense mutations to a presenting phenotype. The presentation of the initial child with dysgammaglobulinemia led us to consider other primary immunodeficiencies, which can present with FHL (22). However, the presence of a sister who carried the identical *UNC13D* mutations with similar NK cell dysfunction but normal immunoglobulins, and displayed an isolated predisposition to severe viral infection, added weight to the idea that this was not a combined immunodeficiency; in addition, *UNC13D* deficiency is documented to potentially cause dysgammaglobulinemia (6). Could we be missing other FHL gene mutations? Recent clinical and experimental reports have suggested that mono-allelic mutations affecting multiple genes in the granule exocytosis cytotoxicity pathway may predispose an individual to atypical FHL (23–25). While mutation screening here did not identify any additional mutations in FHL2-5 causing genes, it is possible that the patients may have inherited polymorphic allele/s in other genes (25) that might have influenced the course of the disease. In principle, the conclusive (and formal) evidence of the disease-causing nature of the *UNC13D* or any other potential disease-causing mutations will require a direct analysis of their function (26). Depending on the gene involved, such studies may be more or less feasible, (e.g., for technical reasons the analysis of *UNC13D* mutations is still in its infancy). Having said that, in the current study, the previously reported case of homozygous p.Arg414Cys pathogenicity (8), the invariable conservation of p.Arg414 and p.Ala918, the extreme rarity of both mutations, the uniform *in silico* prediction of pathogenicity of the mutations, and the impaired degranulation which is corrected posttransplant (Figure 2), all strongly suggests that FHL was due to the bi-allelic mutations in *UNC13D* outlined above.

Here, the assessment of NK cell function in a healthy patient with a known *UNC13D* defect and comparison to an atypically presenting geno-identical sibling have provided several novel insights (Figure 2). The residual NK cell cytotoxic activity assessed prospectively in Patient 2 (when she was healthy) at 20% of control was sufficient for her to avoid disease in early life, but insufficient to protect her when she encountered EBV. This supports data from chimeric post stem cell transplant FHL patients where up to 30% of “normal” donor cells may be needed to prevent HLH recurrence (27). This notion is also consistent with the findings of mono-allelic inheritance of FHL-causing mutations affecting cytotoxic lymphocyte function (10). Some studies suggested that mono-allelic mutations, which reduce cytotoxicity may also predispose to various cancers (4, 28) and to the occurrence of macrophage activation syndrome in patients with systemic juvenile idiopathic arthritis (21).

Another important observation relates to immune perturbations that occur in patients with FHL. On the one hand, the geno-identical sisters had virtually identical levels of degranulation per cell based on mean fluorescence intensity of CD107a expression in activated NK cells. However, Patient 1, who was chronically inflamed and had a perturbed immune system as manifested by B-cell lymphopenia and dysgammaglobulinemia, had undetectable NK cell cytotoxicity, even though she might have been expected to have activity similar to her asymptomatic sister. Importantly, we formally demonstrated that active HLH appeared to aggravate NK dysfunction in Patient 2, although the molecular mechanism remains unclear.

## Concluding Remarks

Despite delayed onset, patients with hypomorphic genotypes can present with or progress to aggressive HLH or hematological malignancies, with case series demonstrating poor outcomes for such patients (6). It is therefore important to consider functional and genetic screening for FHL in older children with any of a gamut of pervasive atypical inflammatory presentations or where “red flags” such as dysgammaglobulinemia are present.

## Ethics Statement

This study was carried out in accordance with the recommendations of Sydney Children’s Hospital ethics committee with written informed consent from all subjects. All subjects gave written informed consent in accordance with the Declaration of Helsinki. The protocol was approved for CIRCA—investigation of primary immunodeficiencies, by the Sydney Children’s Hospital.

## Author Contributions

PG, JT, and IV designed the study and co-wrote a manuscript; PG, BS, SR, RM, MB, KG, IA, and EK assessed, diagnosed, and treated the patients; IV and KT conducted experiments.

## Conflict of Interest Statement

The authors declare that the research was conducted in the absence of any commercial or financial relationships that could be construed as a potential conflict of interest.
